# Cysteine and glycine-rich protein 3 (Crp3) as a critical regulator of elastolysis, inflammation, and smooth muscle cell apoptosis in abdominal aortic aneurysm development

**DOI:** 10.3389/fphys.2023.1252470

**Published:** 2023-12-19

**Authors:** Ana Barbosa Marcondes de Mattos, Joao Carlos Ribeiro-Silva, Miriam Helena Fonseca-Alaniz, Iuri Cordeiro Valadão, Erasmo Simão da Silva, Jose Eduardo Krieger, Ayumi Aurea Miyakawa

**Affiliations:** ^1^ Laboratorio de Genética e Cardiologia Molecular, Instituto do Coração (InCor), Faculdade de Medicina da Universidade de São Paulo, São Paulo, Brazil; ^2^ Divisão de Cirurgia Vascular e Endovascular, Departamento de Cirurgia, Faculdade de Medicina da Universidade de São Paulo, São Paulo, Brazil

**Keywords:** abdominal aortic aneurysm, cysteine and glycine-rich protein-3, smooth muscle cell, apoptosis, elastolysis

## Abstract

Abdominal aortic aneurysm (AAA) is a life-threatening vascular disease for which surgical or endovascular repair are the only currently available therapeutic strategies. The development of AAA involves the breakdown of elastic fibers (elastolysis), infiltration of inflammatory cells, and apoptosis of smooth muscle cells (SMCs). However, the specific regulators governing these responses remain unknown. We previously demonstrated that Cysteine and glycine-rich protein 3 (Crp3) sensitizes SMCs to apoptosis induced by stretching. Building upon this finding, we aimed to investigate the influence of Crp3 on elastolysis and apoptosis during AAA development. Using the elastase-CaCl_2_ rat model, we observed an increase in Crp3 expression, aortic diameter, and a reduction in wall thickness in wild type rats. In contrast, Crp3^−/−^ rats exhibited a decreased incidence of AAA, with minimal or no changes in aortic diameter and thickness. Histopathological analysis revealed the absence of SMC apoptosis and degradation of elastic fibers in Crp3^−/−^ rats, accompanied by reduced inflammation and diminished proteolytic capacity in Crp3^−/−^ SMCs and bone marrow-derived macrophages. Collectively, our findings provide evidence that Crp3 plays a crucial role in AAA development by modulating elastolysis, inflammation, and SMC apoptosis. These results underscore the potential significance of Crp3 in the context of AAA progression and offer new insights into therapeutic targets for this disease.

## Introduction

Abdominal aortic aneurysm (AAA) is a complex vascular disease characterized by the progressive dilation of the abdominal aorta, which can ultimately lead to aortic dissection and rupture (Golledge et al., 2006; Golledge et al., 2009). The current management of AAA, whether ruptured or symptomatic non-ruptured, relies on open or minimally invasive surgical procedures (IMPROVE Trial Investigators, 2017; Chaikof et al., 2018). However, these approaches still carry a high risk of morbidity and mortality (Norman et al., 1998; Patel et al., 2016), underscoring the need for improved therapeutic strategies for the prevention and treatment of AAA.

Chronic inflammation of the aorta is a key mechanism associated with AAA development (Lindeman et al., 2011; Meng et al., 2014; Dale et al., 2015). Infiltrating monocytes and macrophages release enzymes such as matrix metalloproteinases (MMPs) and serine proteases, which contribute to the breakdown of elastic fibers (known as elastolysis), a crucial component of the vascular extracellular matrix (ECM) (Pearce and Koch, 2006; Folkesson et al., 2007). This interplay between inflammation, elastolysis, and oxidative free radicals leads to dysfunction and apoptosis of smooth muscle cells (SMCs), further weakening the aortic wall (Thomas et al., 2006; Wang et al., 2015; Cao et al., 2017; Li et al., 2017; Sakalihasan et al., 2018).

We have previously demonstrated that absence of the cysteine and glycine-rich protein 3 (Crp3) in SMCs prevents apoptosis (Campos et al., 2009; Campos et al., 2018), promoting SMCs accumulation and the ultimate occlusion of vein grafts (Campos et al., 2009; Campos et al., 2018). Crp3 belongs to the cysteine and glycine-rich protein (CRP) family of LIM-only proteins, which play a role in coordinating SMC differentiation, migration, and survival by promoting the formation of macromolecular signalosomes (Chang et al., 2003; Weiskirchen and Günther, 2003; Wang et al., 2006; Chen et al., 2013). We also demonstrated that Crp3 is constitutively expressed by arterial SMCs, while its expression is sensitive to stretch in vein SMCs (Campos et al., 2009), suggesting that it may be required to the vascular response to mechanical damage. However, to this date, evidence of the Crp3 participation in AAA pathogenesis is lacking. Gene expression analyses have shown differential expression of the Crp3 paralogs Crp1 and Crp2 in aneurysmal disease (Hinterseher et al., 2012; Matsumoto et al., 2014) and the absence of Crp2 was recently found to prevent angiotensin II-induced AAA in mice (Chen et al., 2022). However, the expression and functional impact of Crp3 in AAA, as well as its potential role in linking elastolysis, SMC inflammation, and apoptosis during AAA development, remain to be elucidated.

In this study, we show evidence demonstrating the critical role of Crp3 expression in the development of experimental AAA in rats. We observed an upregulation of Crp3 expression in rat and human AAA samples, whereas Crp3 knockout attenuated SMC apoptosis and elastolysis in the abdominal aorta, resulting in reduced incidence and development of AAA in rats.

## Materials and methods

### Human tissue collection

Normal aortic samples were obtained from patients undergoing aortocoronary bypass surgery at the Heart Institute, while human abdominal aortic aneurysm samples were collected from patients undergoing open repair surgery at the Hospital das Clínicas, Faculdade de Medicina da Universidade de São Paulo. The samples were placed in 4% paraformaldehyde and embedded in paraffin for histological processing.

### Rat model of abdominal aortic aneurysm (AAA)

The Crp3^−/−^ rat was generated on a Wistar background, where pairs of custom CompoZr^®^ Zinc Finger Nucleases (Sigma-Aldrich, St. Louis, MO) targeting the Csrp3 gene generated a 4-bp frameshift deletion (deleted CATGC and inserted A) overlapping the start codon in exon 1, as previously described (Campos et al., 2018). Male wild type and Crp3^−/−^ rats (10–12 weeks old, 250–350 g) were submitted to intraperitoneal injection of heparin (70 UI/kg) and anesthetized with ketamine (50 mg/kg) and xylazine (10 mg/kg). After anesthesia, a midline section was made to expose the abdominal cavity. The bladder and the intestines were suspended and kept moist with water-soaked gauze during the entire procedure. The infrarenal segment of the abdominal aorta was exposed, clamped in its extremities and AAA was induced with intraluminal filling with porcine pancreatic elastase (1 U/μL, EMD Millipore 324682) and extraluminal exposure to 0.5 M calcium chloride (CaCl_2_), as previously described in the literature (Tanaka et al., 2009). The control animals underwent to the same surgical procedures as AAA animals and saline solution was used instead of elastase and CaCl_2_. After 20 min of exposure, the clamps were removed to restore blood flow, the bladder and intestines were returned to the abdominal cavity and the incision was closed with a 5.0 nylon suture. The animals were kept warm in a recovery cage until recovery from anesthesia. All animal procedures were in accordance with the Heart Institute guidelines for the use and care of laboratory animals (SDC 4398/16/064 CEUA 080/16).

### Aortic diameter measurements

The abdominal aortic diameter of saline and AAA rats was determined in a high-resolution ultrasound system set to perform two-dimensional imaging (B Mode) with a real time microvisualization scanhead (RMV 704), central frequency of 40 MHz (30 Hz frame rate), focal length of 6 mm and field of view of 8 mm × 8 mm (Visualsonics). Briefly, rats anesthetized with a mixture of xylazine and ketamine were laid supine on a heated table, and ultrasound transmission gel was applied to their abdomen. Short-axis scans of the abdominal aorta from the region between the left renal arterial branch and the infrarenal regions were taken before aneurysm surgery and 3, 7 and 14 days after Elastase-CaCl_2_-induced AAA. Lumen diameter enlargement was expressed as dilation ratio % = [(AAA diameter—aortic diameter)/aortic diameter] × 100. AAA was defined as aortic dilation above 50% (Johnston et al., 1991).

### Histopathologic analyses

Fourteen days after AAA induction, the rats were killed with sodium pentobarbital overdose (200 mg/kg), aortic segments were harvested, washed with a heparinized saline solution, fixed by pressure perfusion with 4% paraformaldehyde and embedded in paraffin for the downstream histomorphological analysis. To evaluate collagen and elastin content, 3 µm-thick transverse sections were stained with picrosirius red and Verhoeff’s van Gieson, respectively. The evaluation of apoptotic events was performed with Click-iT™ Plus TUNEL Assay Kits for *In Situ* Apoptosis Detection, following the manufacturer’s instructions. Tissue treated with DNase I to induce TUNEL-positive DNA strand breaks was used as positive control. The negative control samples were not incubated with TdT reaction mixture (Supplementary Figure S1A).

For the immunohistochemistry assays, rat and human (when stated) aortic sections were deparaffinized in citrisolv^®^, dehydrated in serial alcohol dilutions and submitted to antigen retrieval in citrate buffer, pH 6.0 at 90°C for 40 min. Blocking of endogenous peroxidase and unspecific binding was performed with hydrogen peroxide and bovine serum albumin, respectively. Sections were incubated overnight in the presence rabbit anti-Crp3 (Santa Cruz Biotechnology sc-98827 1:100), succeeded by rabbit HRP-conjugated secondary antibody (Invitrogen, 1:1,000) and counterstain with Meyer’s hematoxylin. The stained sections were examined by light microscopy and quantified with aid of the Leica Qwin Software (Leica). Heart samples were used as Crp3-positive reaction. Heart and aorta negative controls were not incubated with antibody (Supplementary Figure S1B).

The detection of macrophages in aneurysm lesions was performed by incubating rat aortas with a primary antibody against CD68 (Abcam ab49777 1:50) and subsequent incubation with Alexa fluor 555-conjugated secondary antibody (Molecular Probes, 1:250) and DAPI (1:1,000, Molecular Probes) for 2 h. The binding reaction was performed with Histofine^®^ (Nicherei Bioscience). Fluorescent sections were imaged in the Zeiss Axio Observer Z1 Microscope with an air objective of ×20 and light source intensity of 58.32%. The number of CD68^+^ nuclei was quantified with Image J.

### SMC apoptosis and Mmp measurements

Primary wild type and Crp3^−/−^ aortic SMCs were obtained via explant, as previously described (Campos et al., 2018). Shortly, aortic segments were cleaned off the adventitia and intima layer, cut in small fragments and plated on gelatin-coated six-well plates. The fragments were allowed to adhere and cultured in Dulbecco’s Modified Eagle’s Medium (DMEM) (LGCbio) supplemented with 20% FBS (Gibco), 100 U/mL penicillin (Gibco), and 100 μg/mL streptomycin (Gibco). SMCs derived from aortic fragments were isolated and expanded up to passage 3. Apoptosis evaluation was performed in serum-starved wild type and Crp3^−/−^ aortic SMCs exposed to 100 μM N-acetyl-D-sphingosine (Ceramide; Sigma A9171) for 24 h, then stained with propidium iodide and fluorescein isothiocyanate–annexin V (Molecular Probes). The detection of SMCs with hypodiploid nuclei was performed in a FACSCalibur flow cytometer and analyzed with the aid of the Cell Quest software (Becton Dickinson). Data expressed as the % of propidium iodine negative/annexin V positive cells. Around twenty thousand events were considered in all assays.

To evaluate the secretion of Mmps and their inhibitor Timp-2, cells were serum starved for 24 h, then stimulated with 10 ng/mL of Interleukin (IL)-1β (IL-1β, Peprotech) for other 24 h. After this period, cell media was collected and Timp-2 (Abcam ab213923), Mmp-2 (GE Healthcare RPN2631) or Mmp-9 (GE Healthcare RPN2634) secretion and/or activity were analyzed with specific immuno-capture assays following the manufacturer’s instructions. Following media collection, total cell protein was lysed in ice-cold RIPA buffer (50 mM NaCl, 50 mM Tris/HCl, pH 7.4, 1 mM EDTA, 1% Triton X-100, 0.1% NP-40, 1 mM PMSF, protease and phosphatase inhibitors’ cocktails) and protein concentration was determined with the Pierce Assay (Thermo Scientific) to further normalize total and active Mmp levels.

### SMCs gelatinase/collagenase and elastase activity assay

The gelatinolytic/collagenolytic and elastolytic activities of SMCs were measured by means of the EnzChek gelatinase/collagenase and elastase assay kits (E12055 and E12056, respectively, Molecular Probes, United States), following the manufacturer’s instructions. Briefly, cells at 100% confluence in a 96-wells plate were serum starved for 24 h, then stimulated with 10 ng/mL of Interleukin-1β (IL-1β, Peprotech) for other 24 h. After this period, the cells were washed with 1x phosphate-buffered saline (PBS; pH 7.4) and fixed using 100% methanol at −20°C for 15 min. The cells were incubated with DQ gelatin at a 100 μg/mL or DQ elastin at 25 μg/mL concentration for 2 h, light protected at room temperature. The nuclei were next stained with DAPI. Images were acquired on the EVOS M7000 imaging system (Thermo Fisher Scientific, United States), and image analysis (mean fluorescence intensity in the cytoplasm) was performed using CellProfiler v.4.1.3.

### Macrophage harvesting and culture

Wild type and Crp3^−/−^ macrophages were prepared by collecting bone marrow cells from femurs, as previously described (Zogbi et al., 2020). The cell suspension was passed through a 100 µm-nylon cell strainer (BD Falcon), collected by centrifugation at 1200 RPM for 5 min at 4°C, and resuspended in RPMI medium containing 10% FBS, 100 U/mL penicillin, 100 mg/mL streptomycin. The cells were then cultured in the presence of macrophage colony stimulating factor (30 ng/ml M-CSF; Peprotech) for 7 days. After this period, macrophages were stimulated with M-CSF combined with either interferon-γ (10 ng/mL; Peprotech) or interleukin-4 (20 ng/mL; Peprotech) for the classical (M1) or alternative (M2) polarization, respectively. 48 h post-stimulation, the cells were lysed with Trizol reagent (Invitrogen) for downstream expression analysis.

### cDNA synthesis and real-time RT-PCR

Total RNA was isolated from polarized macrophages using Trizol reagent (Invitrogen) and cDNA was synthetized with SuperScript III Reverse Transcriptase (Invitrogen) according to the manufacturer’s specifications. 100 ng of cDNA were used for the RT-PCR reaction (SYBR Green PCR Master Mix-PE Applied Biosystems), performed on the ABI Prism 7700 Sequence Detection System (Applied Biosystems). The list of the oligonucleotide primers used can be found in Table 1. Analysis of target gene expression was assayed in triplicate and the comparative threshold (C_T_) cycle method was used for data analysis.

**TABLE 1:** List of oligonucleotide primers used in the real time RT-PCR.

Gene	Forward oligonucleotide	Reverse oligonucleotide
*Gapdh*	ATGGTGAAGGTCGGTGTG	GAA​CTT​GCC​GTG​GGT​AGA​G
*Mmp2*	AGA​AGG​CTG​TGT​TCT​TCG​CA	AAA​GGC​AGC​GTC​TAC​TTG​CT
*Mmp9*	CCT​GGA​ACT​CAC​ACA​ACG​TC	CCG​GTT​GTG​GAA​ACT​CAC​AC
*Timp2*	ACA​TCT​ATG​GCA​ACC​CCA​TCA	GGG​GGC​CGT​GTA​GAT​AAA​TTC

### Statistical analysis

Data expressed as mean ± standard deviation. Data normality was assessed by the Shapiro–Wilk test or the Kolmogorov–Smirnov test. When the interaction between genotype (wild type versus Crp3^−/−^) and treatment (saline versus AAA; control versus IL-1β; DMSO versus Ceramide) was evaluated, two-way ANOVA followed by Tukey’s *post hoc* test was employed. Genotype × Genotype or Normal × AAA comparisons were made with Student’s t-test. *p* values < 0.05 were considered statistically significant.

## Results

### Absence of Crp3 prevents abdominal aortic aneurysm development and protects SMCs from apoptosis

**FIGURE 1:**
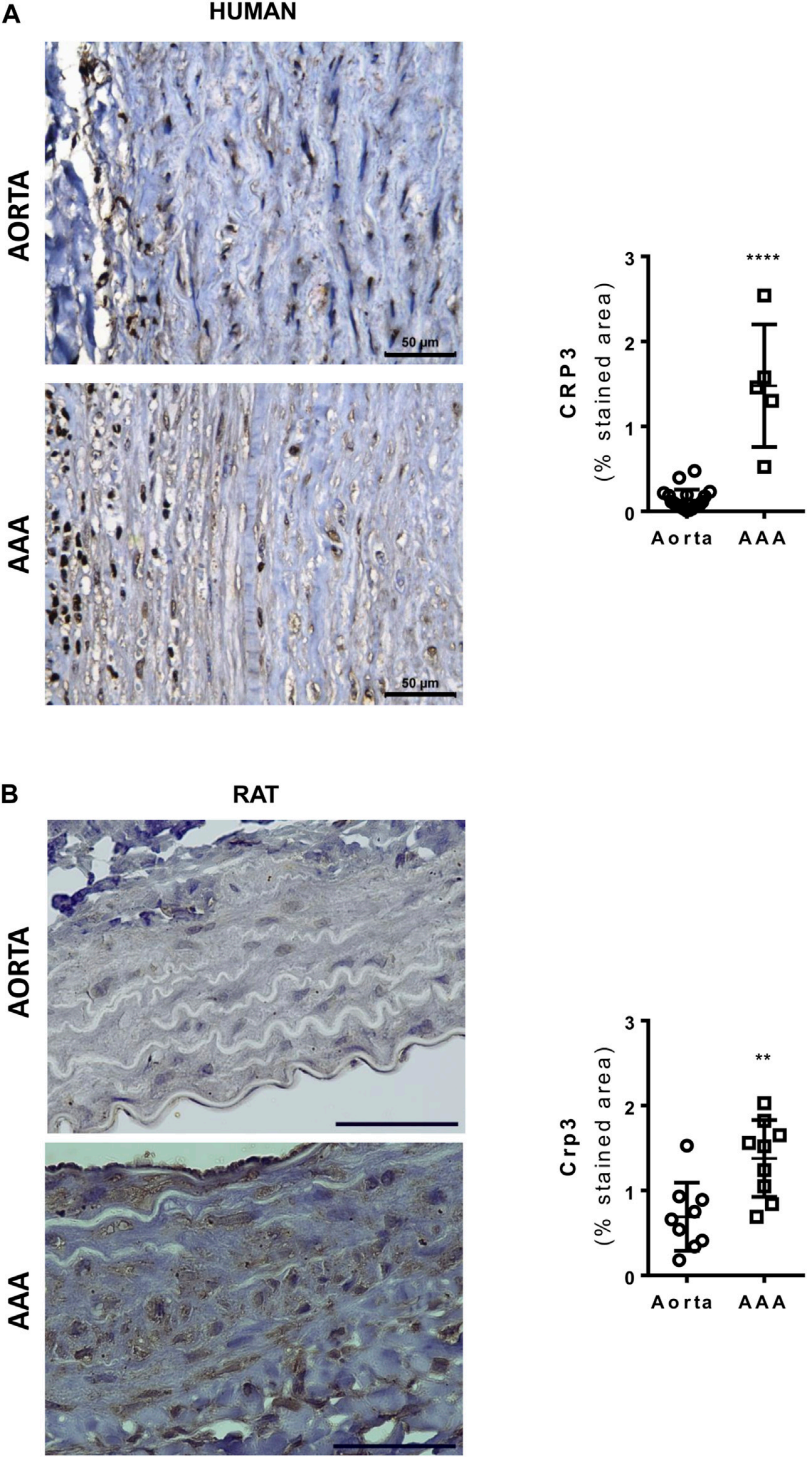
Crp3 expression is induced in human and rat abdominal aortic aneurysm (AAA). **(A)** Representative images of Crp3 staining (brown) in human AAA. The quantification shows modulation of Crp3 in human AAA (*n* = 5) compared to normal aorta (*n* = 12). **(B)** Representative images of Crp3 staining (brown) in rat AAA. Similarly, Crp3 expression increases after 14 days of elastase-calcium chloride induction of AAA in rats (*n* = 9). The black scale bar represents 50 μm. **** indicates *p* < 0.0001 and ** indicates *p* = 0.0036.

**FIGURE 2:**
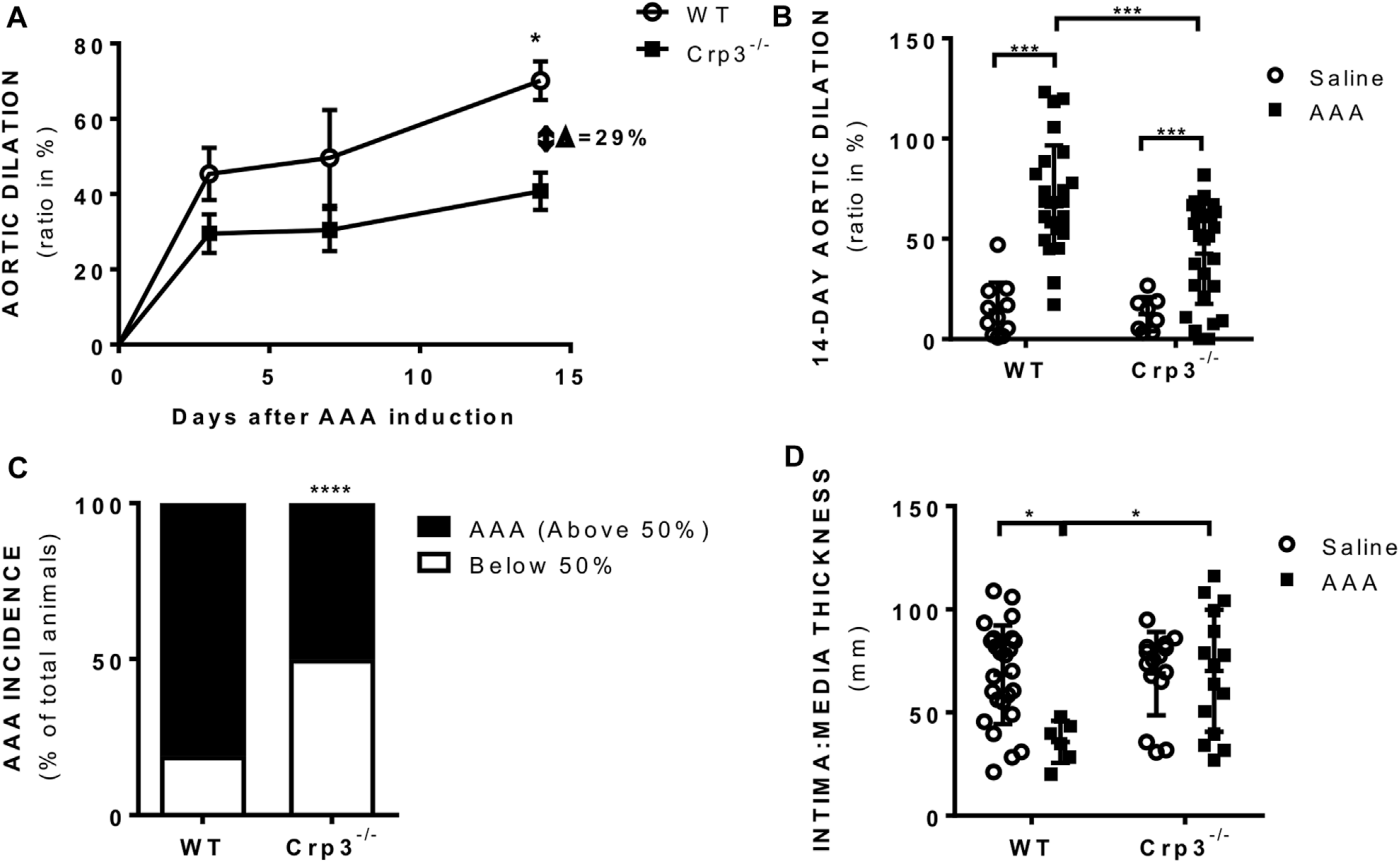
Crp3 absence prevents AAA development. **(A)** Aortic diameter analysis performed 3 days (*n* = 10–14), 7 days (*n* = 5–9) and 14 days (*n* = 26–28) after elastase-CaCl_2_-AAA induction, showing increase in aortic diameter in AAA compared to saline in WT rats, which was attenuated in Crp3^−/−^ AAA. * indicates *p* = 0.020. **(B)** Depiction of the increase in aortic lumen diameter 14 days after AAA induction in WT and Crp3^−/−^ rats and **(C)** graphical representation of the lowered AAA incidence in Crp3^−/−^ rats in comparison with WT AAA. WT (*n* = 26) and Crp3^−/−^ (*n* = 27) *** indicates *p* < 0.001 and **** indicates *p* < 0.0001. **(D)** AAA development in WT rats was associated with reduction in intima: media thickness, while no change was observed upon AAA induction in Crp3^−/−^ rats. WT saline (*n* = 25), Crp3^−/−^ saline (*n* = 15), WT AAA (*n* = 6), Crp3^−/−^ AAA (*n* = 15). * indicates *p* = 0.014.

Histopathological analysis revealed that Crp3 expression was comparably induced in human and rat abdominal aortic aneurysm (AAA) samples (Figures 1A, B). Interestingly, in both human and rat AAA samples, Crp3 expression was no longer confined to the media layer, but present across the entire vascular wall. To investigate the impact of Crp3 on AAA, we utilized the elastase/CaCl_2_ AAA model (Tanaka et al., 2009). We observed progressive aortic dilation in wild-type (WT) rats (Figure 2A), resulting in a 73% increase in lumen diameter after 14 days (Figure 2B). In contrast, Crp3^−/−^ rats exhibited only a 44% lumen enlargement with attenuated AAA incidence (Figure 2C), indicating protection against AAA development. Consistent with these findings, vessel wall thickness decreased in WT rats, whereas Crp3^−/−^ abdominal aorta wall thickness remained unchanged (Figure 2D).

**FIGURE 3:**
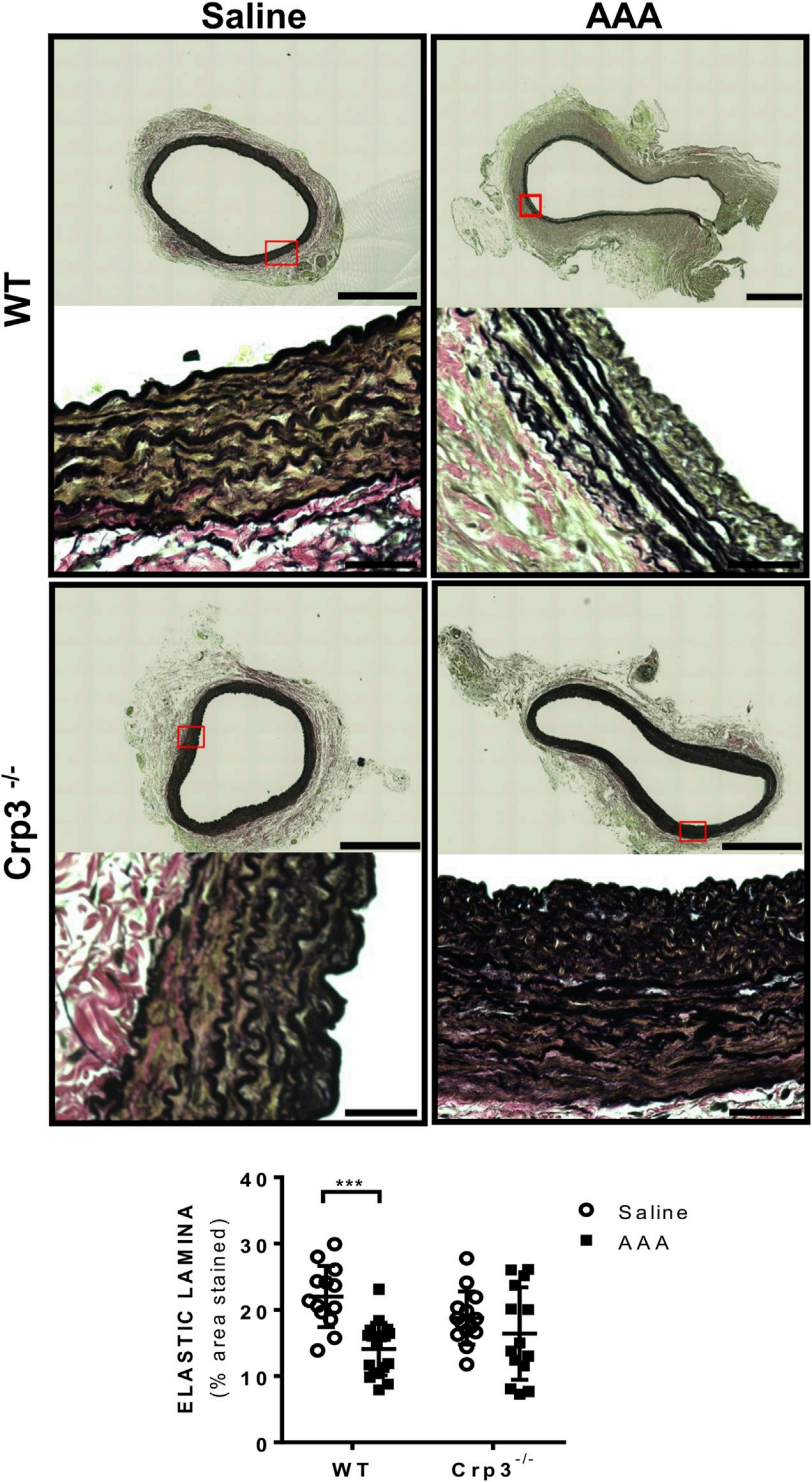
Absence of elastolysis in response to AAA induction in Crp3^−/−^ rats. Verhoeff’s van Gieson staining depicting elastic fibers degradation in WT AAA vs. saline but no difference between saline and AAA in Crp3^−/−^ animals, as shown in the quantification. WT saline (*n* = 13), Crp3^−/−^ saline (*n* = 15), WT AAA (*n* = 18), Crp3^−/−^ AAA (*n* = 14). The black scale bar represents 500 μm (images at lower magnification or 30 μm (images at higher magnification). *** indicates *p* = 0.0002.

**FIGURE 4:**
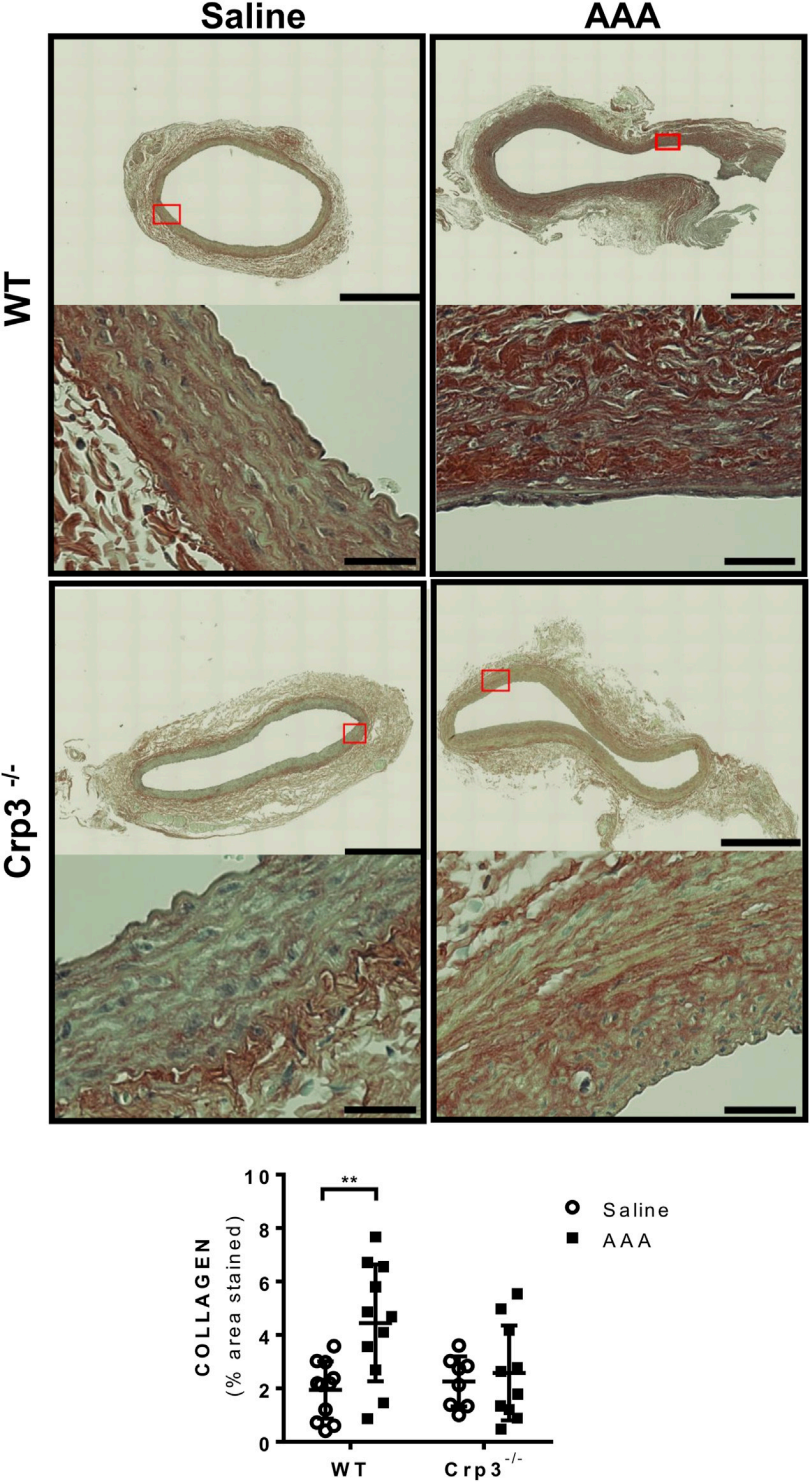
Absence of collagen deposition in response to AAA induction in Crp3^−/−^ rats. Collagen amount evaluation via Picrosirius Red staining, demonstrating that WT AAA development increases collagen, while Crp3^−/−^ remains unchanged. WT saline (*n* = 11), Crp3^−/−^ saline (*n* = 8), WT AAA (*n* = 11), Crp3^−/−^ AAA (*n* = 10). The black scale bar represents 500 μm (images at lower magnification or 30 μm (images at higher magnification). ** indicates *p* = 0.0093.

Considering the crucial role of elastolysis in AAA, we initially assessed the baseline levels of elastic fiber content associated with genetic ablation of Crp3 and observed no changes compared to WT animals (Figure 3). AAA induction resulted in reduced elastic fibers in WT rats but not in Crp3^−/−^ rats (Figure 3). Collagen content increased in WT aortas, whereas it remained unchanged in Crp3^−/−^ aortas, suggesting a compensatory collagen response to the decreased elastic fibers in WT (Figure 4).

**FIGURE 5:**
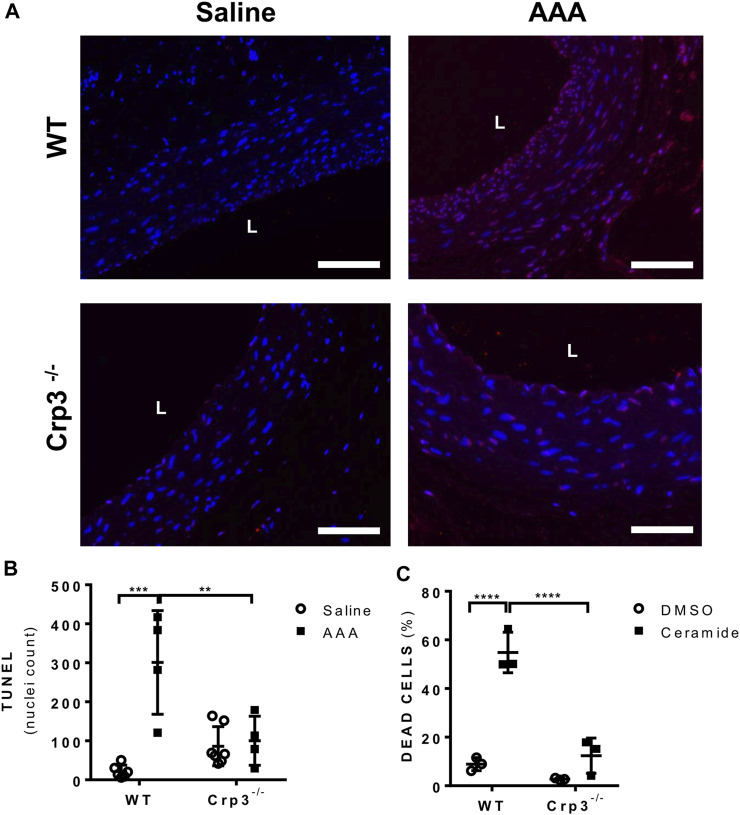
Absence of Crp3 protects SMCs from apoptosis. **(A)** Representative images of TUNEL assay demonstrating the presence of apoptosis in WT AAA but not in Crp3^−/−^. The white scale bar represents 50 μm. **(B)** Quantitative analysis of TUNEL staining performed in saline and elastase-CaCl_2_ aortas of WT and Crp3^−/−^ rats for detection of apoptotic nuclei. AAA development was accompanied of apoptosis in WT AAA but not in Crp3^−/−^ AAA. WT saline (*n* = 6), Crp3^−/−^ saline (*n* = 7), WT AAA (*n* = 4), Crp3^−/−^ AAA (*n* = 4). ** indicates *p* = 0.043 and *** indicates *p* = 0.0002. **(C)** Quantification of apoptosis in isolated aortic SMCs. While WT cells (*n* = 3) showed apoptotic response to 100 µM ceramide, little or no apoptosis is detected in Crp3^−/−^ SMC (*n* = 3). **** indicates *p* < 0.0001.

SMC apoptosis is a hallmark of AAA development (Cao et al., 2017) and Crp3 was previously implicated as a sensitizer of SMCs to apoptosis (Wang et al., 2006; Campos et al., 2018). The absence of Crp3 abolished the apoptotic events observed in WT AAA, as demonstrated by TUNEL labeling of aortic specimens (Figures 5A, B). Indeed, in response to ceramide (an inducer of the mitochondrial apoptotic pathway), cultured Crp3^−/−^ aortic SMCs demonstrated reduced apoptosis when compared to WT SMCs, as assessed by annexin V staining (Figure 5C). This evidence is consistent with our previous observations in SMCs from jugular vein (Campos et al., 2018) and indicates that the absence of Crp3 prevents AAA development, at least in part, by protecting aortic SMCs from apoptosis.

### Impairment of inflammation-induced gelatinase and elastase activity in Crp3^−/−^ SMCs

**FIGURE 6:**
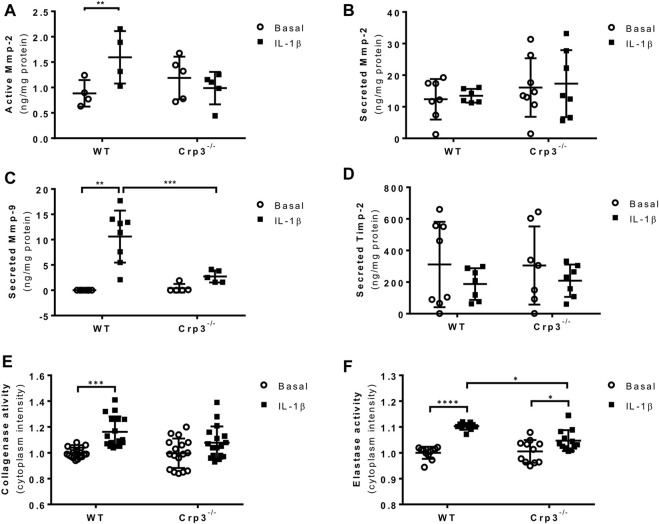
The absence of Crp3 blunts the proteolytic profile of aortic SMCs. **(A)** Quantitative analysis of the secretion and activation of Mmp-2 in the media of control and IL-1β-stimulated aortic SMCs. IL-1β increased Mmp-2 activation, a response that was attenuated in Crp3^−/−^ SMCs (WT *n* = 4, Crp3^−/−^ *n* = 5). ** indicates *p* = 0.027 **(B)** Total Mmp-2 levels remained unchanged (WT *n* = 6, Crp3^−/−^ *n* = 7). **(C)** The secretion of Mmp-9 was induced by IL-1β in WT, but not in Crp3^−/−^ SMCs (WT *n* = 8, Crp3^−/−^ *n* = 5). ** indicates *p* = 0.0048, *** indicates *p* < 0.001 **(D)** Secreted Timp-2 levels remained unchanged in response to IL-1β in WT and Crp3^−/−^ SMCs (WT *n* = 7, Crp3^−/−^ *n* = 7). **(E)** The collagenase activity was induced by IL-1β in WT, but not in Crp3^−/−^ SMC (WT *n* = 17, Crp3^−/−^ *n* = 17). *** indicates *p* = 0.0009. **(F)** The elastolytic activity was induced by IL-1β in both WT and Crp3^−/−^ SMC, but in a higher level in WT (WT *n* = 11, Crp3^−/−^ *n* = 11). * indicates *p* = 0.0208, and **** indicates *p* < 0.0001.

The early stages of AAA are driven by inflammation, leading to the activation of proteases and apoptosis of SMCs, resulting in extracellular matrix (ECM) degradation and aortic wall thinning. To better understand how the absence of Crp3 prevents AAA development, we treated SMCs with IL-1β, one of the main cytokines found at aneurysm lesions (Johnston et al., 2013). IL-1β treatment induced the activation of Mmp-2 in WT SMCs but failed to affect Crp3^−/−^ SMCs (Figure 6A), while total Mmp-2 levels remained unchanged in both groups (Figure 6B). The activity of Mmp-9 could not be detected. However, IL-1β induced the secretion of Mmp-9 in WT SMCs but not in Crp3^−/−^ SMCs (Figure 6C). Secretion of Timp-2, an inhibitor of Mmp activity, was not influenced by IL-1β exposure in both genotypes (Figure 6D). As both Mmp-2 and Mmp-9 are known to cleave collagen and elastin, these observations indicate that the activity of Mmp-2 and secretion of Mmp-9 induced by IL-1β are impaired in Crp3^−/−^ SMCs. Consequently, IL-1β did not increase the gelatinase activity in Crp3^−/−^ cells (Figure 6E) and elastolysis induced by IL-1β in WT SMCs is reduced in Crp3^−/−^ SMCs (Figure 6F).

### The absence of Crp3 promotes elastoprotective macrophage gene expression

**FIGURE 7:**
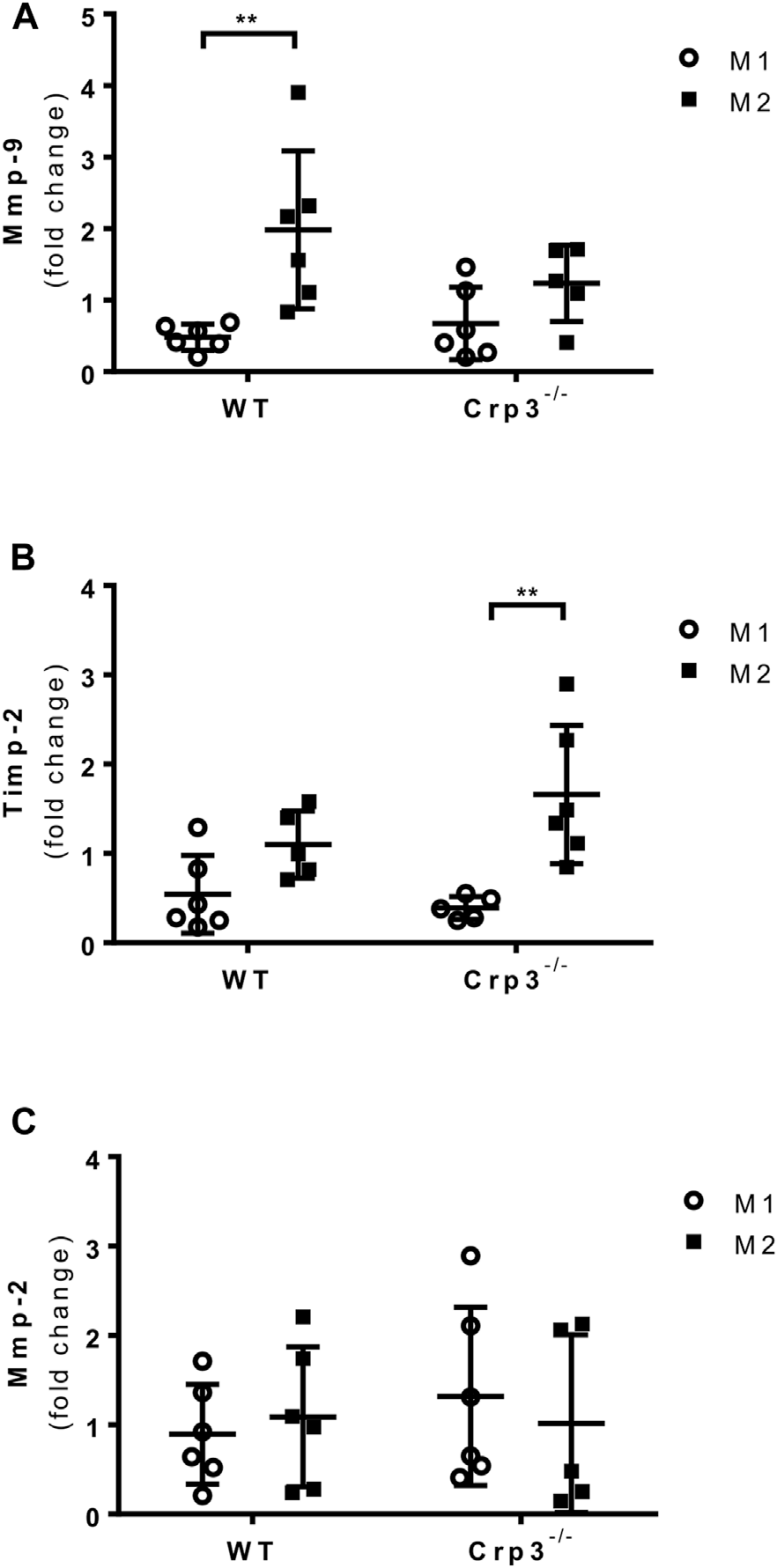
Impact of Crp3 knockout in the inflammatory response. Gene expression analysis showing **(A)** increased expression of Mmp-9 only by wild type macrophages in response to M2 polarization, while **(B)** the expression of Timp-2 is upregulated in Crp3^−/−^, but not in wild type macrophages. **(C)** Mmp-2 expression levels are similar in both groups (WT *n* = 6, Crp3^−/−^ *n* = 5). ** indicates *p* = 0.0017.

Since infiltrating inflammatory cells in human and rat AAA demonstrated Crp3 expression (Supplementary Figure S2A), we quantified CD68^+^ cells to evaluate the presence of macrophages. As expected, we found no staining in normal aortas. Upon AAA induction, there was a surge in CD68^+^ cells that was comparable in wild type and Crp3^−/−^ rats (Supplementary Figure S2B). Macrophages have the ability to transition between classical (M1) and alternative (M2) phenotypes, so we examined whether there was a change in the phenotype of bone marrow-derived macrophages from Crp3^−/−^ animals that could contribute to their protection against AAA. Polarization of primary macrophages towards the alternative (M2-like) phenotype was accompanied by Mmp-9 expression (Figure 7A), with no counterbalance from the Mmp inhibitor Timp-2 (Figure 7B). In contrast, M2-like Crp3^−/−^ macrophages exhibited no upregulation of Mmp-9 but showed increased expression of Timp-2 (Figures 7A, B). Mmp-2 expression levels remained unchanged in both groups (Figure 7C). There were no changes in the expression of genes used as markers of polarized phenotypes in wild-type vs. Crp3^−/−^ macrophages (Supplementary Figure S3). Taken together, these findings suggest that the modulation of the inflammatory status of aortic SMCs and macrophages may synergistically act to prevent the establishment and progression of AAA in Crp3^−/−^ rats.

## Discussion

We explored, for the first time, the role of the Crp3 and its underlying mechanisms involved in the development of AAA. Crp3 showed pleiotropic influence on key cellular processes including SMCs apoptosis, inflammatory response and proteolytic activity and may be explored as a novel therapeutic target to AAA.

We showed that Crp3 expression increases in response to AAA in human samples, similarly to previously described regulation of Crp1 and Crp2 (Crp3 paralogs) in abdominal and thoracic aorta aneurysms (Hinterseher et al., 2012; Matsumoto et al., 2014). These findings corroborate previous evidence from our group and others demonstrating the role of Crp3 in the vascular stress response (Wang et al., 2006; Campos et al., 2009; Campos et al., 2018) and led us to hypothesize that it would impact AAA development. Elastase-CaCl_2_ AAA induction was accompanied of Crp3 induction (which was no longer restrained to the medial vascular layer) increased aortic diameter and reduced wall thickness in WT AAA compared to saline. In contrast, Crp3^−/−^ rats showed attenuated aortic dilation and reduced AAA incidence, demonstrating overall protection against AAA development. The ability of this model to recapitulate the main histopathological aspects of the human AAA is well-documented in the literature (Tanaka et al., 2009) and the findings of Crp3 induction further confirmed its usefulness to dissect the role of Crp3 in AAA pathogenesis.

In the aorta, Crp3 is expressed by SMCs, and in contrast to the terminally differentiated striated muscle cells, aortic SMCs transit between differentiated and dedifferentiated phenotypes in response to changes in their environment, such as the establishment of a local inflammatory response (Bennett et al., 2016). This is confirmed by the diminished expression of SM α-actin upon stimulation with IL-1β (Herring and Smith, 1996; Clément et al., 2007). It is well-established that Crp1 and Crp2 act as cofactors of the Serum responsive factor (SRF), gatekeeper of the SMC transcription machinery (Chang et al., 2003; Alexander and Owens, 2012). In this scenario, taking into account its poor capacity of activating SMC gene promoters (Chang et al., 2003), Crp3 could prevent SMC differentiation mediated by the SRF-GATA-Crp1-Crp2 complex, therefore promoting the inflammatory response in the vicinity of vascular disease. In line with this concept, we observed a blunted inflammatory response in Crp3^−/−^ SMCs stimulated with IL-1β, leading to a reduced proteolytic profile, measured as the secretion and activation of matrix metalloproteinases. Similar effect was observed in the phenotype of bone marrow-derived macrophages isolated from Crp3^−/−^ rats. Despite the similarity in the common aspects of M1 and M2-like macrophage polarization between WT and Crp3^−/−^ macrophages and in their recruitment to aneurysmal lesions, we found that while WT polarization towards the alternative (M2-like) phenotype is accompanied of increased expression of Mmp-9, Crp3^−/−^ M2-like macrophages showed no increase in Mmp-9, but rather upregulation in the expression of the Mmp inhibitor Timp-2, suggesting that there is a common mechanism for Crp3 in the inflammatory state of both, SMC and macrophages. In addition, AAA was associated with Crp3 increase across the entire vascular wall, suggesting that fibroblast and endothelial cell Crp3 expression is also subject to forces and may play a role in AAA pathogenesis.

The underlying mechanism for the effect of Crp3 in the inflammatory response of SMCs remains to be elucidated but we have previously demonstrated that Crp3 interacts with and modulates the activation of the Focal adhesion kinase (Fak) (Campos et al., 2018). Fak is implicated in the SMC inflammatory response via c-Jnk activation, upregulating Monocyte chemoattractant protein-1 (Mcp-1) and Matrix metalloproteinase-2 (Mmp-2) (Varadarajulu et al., 2005; Yamashita et al., 2013). Thus, changes in the Crp3-Fak axis could also impact inflammation and elastolysis during AAA development in the absence of Crp3. In fact, while AAA induction was accompanied of marked elastolysis in WT rats, Crp3^−/−^ showed no change in elastic fibers content. These findings were associated with the inflammatory response of Crp3^−/−^ SMC that, in the presence of IL-1β, displayed reduced secreted Mmp-9 and active Mmp-2 levels, leading to blunted elastolytic activity. A similar behavior for Crp2 was described in breast cancer. Analysis performed in a cohort of over one thousand breast cancer patients showed that Crp2 expression positively correlates with expression of inflammatory genes such as the Urokinase plasminogen activator surface receptor (uPAR), Mmp-2 and Mmp-14 (Hoffmann et al., 2018). Crp2 absence abrogated synthesis and secretion of Mmp-9 and also the Mt1-Mmp-mediated ECM degradation in breast cancer cell lines (Hoffmann et al., 2016; Hoffmann et al., 2018). In SMCs, Chen et al. (2022) recently demonstrated that Crp2 knockout inhibits SMC Erk 1/2-Mmp2 signaling, promoting ECM homeostasis and preventing AAA development in mice.

In addition to the role in inflammation and elastolysis, Crp3 absence also modulated the apoptosis of SMCs, a key event in AAA (Golledge, 2019)**.** We found that Crp3 absence impaired apoptosis in the setting of AAA in rats, as well as in isolated SMC exposed to ceramide. The role of Crp3 in apoptosis is one of the evidences highlighting its singularity among the other two CRPs expressed in the arterial tree, as absence of Crp3 (Wang et al., 2006) but not of Crp1 or Crp2 was able to protect SMCs from apoptosis in response to wire injury (Lilly et al., 2010), thus it should be considered a potential therapeutic target to be explored. Evidences from our group demonstrated that Crp3 role in SMC apoptosis was attributed, at least in part, to its interaction with Fak, therefore affecting integrin-dependent signaling pathways (Campos et al., 2018). Collectively, this evidence allows us to establish a model where Crp3 lies at the interface between the signaling mechanisms coordinating SMC inflammation, elastolysis and apoptosis. In the absence of Crp3, there is an impairment in these mechanisms, ultimately protecting Crp3^−/−^ from AAA development.

Like any study employing experimental models of disease, this study has limitations. While the elastase-CaCl_2_ model successfully reproduces phenomena such aortic dilation, elastin degradation, and inflammatory cell infiltration, it lacks features such as atherosclerosis and intraluminal fibrosis, which are commonly found in human AAA lesions and have a major impact in AAA progression (Golledge et al., 2006; Tanaka et al., 2009). Human AAA is generally fusiform and eventually ruptures, while elastase-CaCl_2_-induced AAA is saccular and does not undergo rupture (Tanaka et al., 2009), differences that explain the lethality of AAA in humans but not in this model. It is well-established that AAA primarily affects males, with prevalence four-to-five times higher in males compared to age-matched females (Katz et al., 1997; Lederle et al., 2001). Some authors may even consider female sex a negative risk for the development of small AAA (Lederle et al., 2000). Nevertheless, AAA in females is small but more aggressive, with high rupture incidence (Hannawa et al., 2009). The Crp3 contribution to AAA development in male and females remains to be elucidated. Finally, while we focused on smooth muscle cells, further research is warranted to understand the mechanisms of Crp3 induction in endothelial cells and fibroblasts during AAA, as well as their contribution to the development and progression of this disease.

Altogether, we provide evidence for a new Crp3 role in the development of abdominal aortic aneurysm. Although further investigation of the mechanisms by which Crp3 expression affects AAA, therapeutic modulation of Crp3 might serve to attenuate inflammation and media layer degeneration, therefore preventing the progression and rupture of abdominal aortic aneurysms.

## Data Availability

The original contributions presented in the study are included in the article/Supplementary Material, further inquiries can be directed to the corresponding author.

## Abbreviations

AAA, Abdominal aortic aneurysm; CRP, Cysteine and glycine-rich proteins family; Crp3, Cysteine and glycine-rich protein 3; ECM, Extracellular matrix; Fak, Focal adhesion kinase; IL-1β, Interleukin-1β; Mmp, Matrix metalloproteinase; SMCs, Smooth muscle cells.
