# Supplementary material for: Cysteine and glycine-rich protein 3 (Crp3) as a critical regulator of elastolysis, inflammation, and smooth muscle cell apoptosis in abdominal aortic aneurysm development
Source: Front Physiol. 2023 Dec 19;14:1252470. doi: 10.3389/fphys.2023.1252470 (PMC10762791; doi:10.3389/fphys.2023.1252470)
Supplement: Supplementary file 1 [file DataSheet1.PDF]

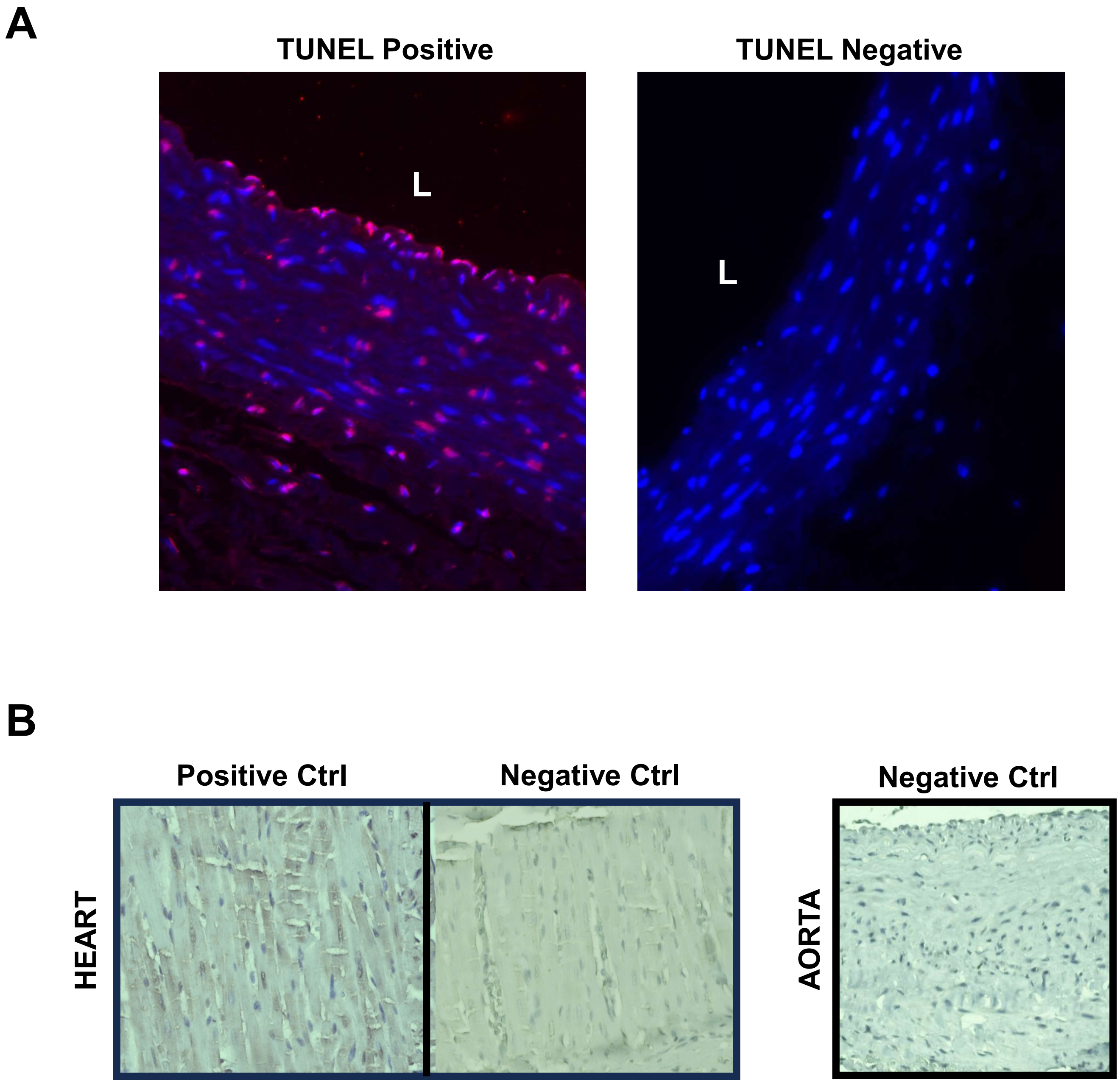

Supplementary Figure 1. Positive and negative controls for histopathological analyses. **(A)** TUNEL-positive control was induced by the treatment of the sample with DNase I. For TUNEL-negative control the sample were not incubated with TdT reaction mixture. **(B)** Heart sample, which is known to express high levels of Crp3, was used as positive control for Crp3 immuhistochemistry. Heart and aorta negative controls were not incubated with the antibody.

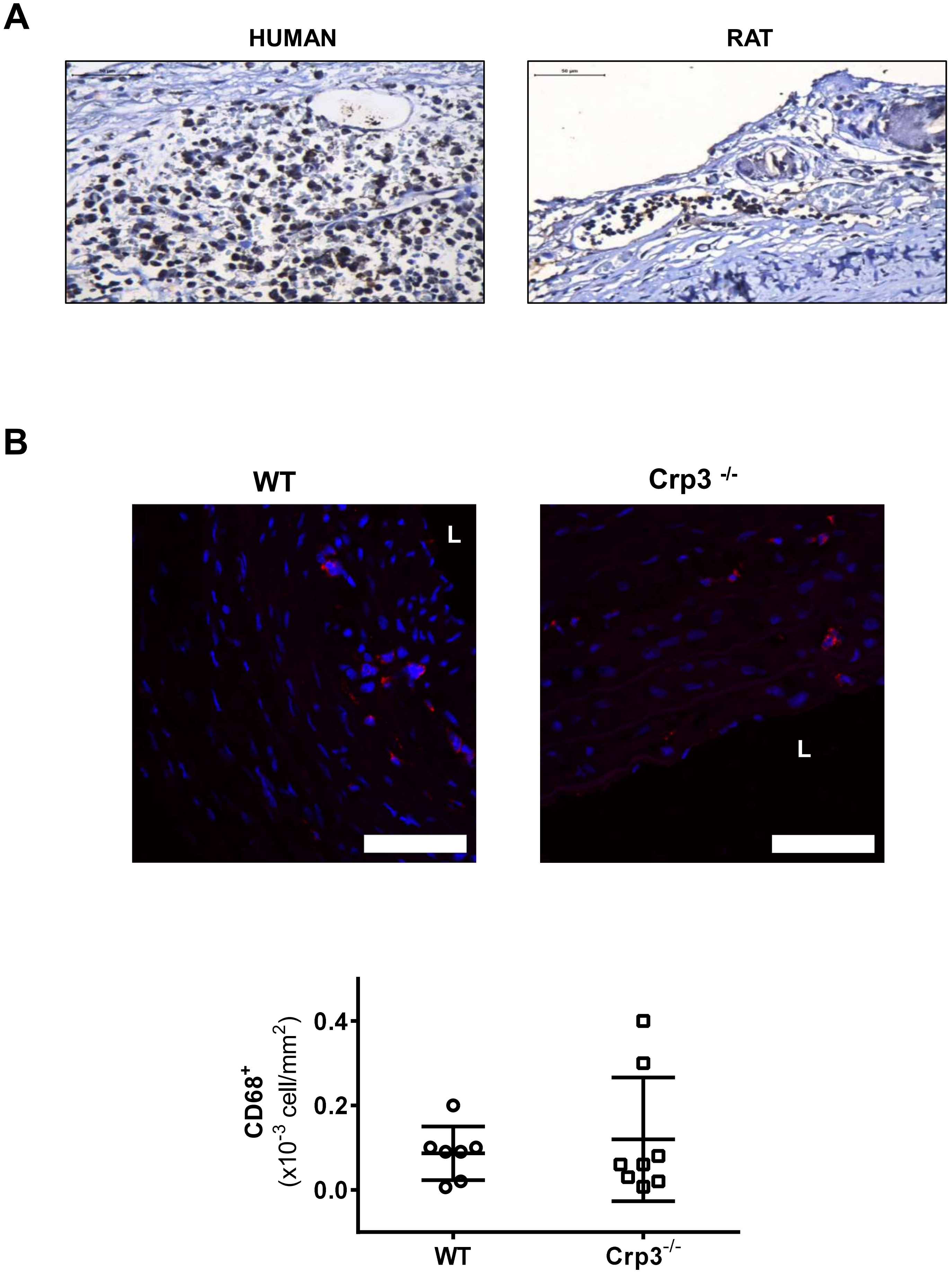

Supplementary Figure 2. **(A)** Immunohistochemistry against Crp3 (brown) demonstrating the expression in infiltrating inflammatory cells in human and rat AAA (human AAA n=5; rat AAA n=9). **(B)** Representative images and quantification of the immunofluorescence against CD68 performed in WT and Crp3$^{-/-}$ aortas showing comparable levels of macrophages. Magnification: 400X (n=6).

A

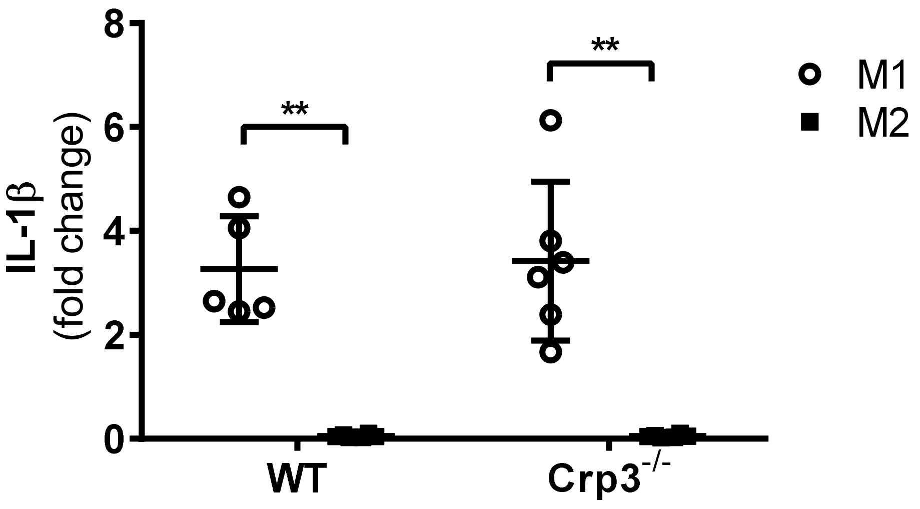

B

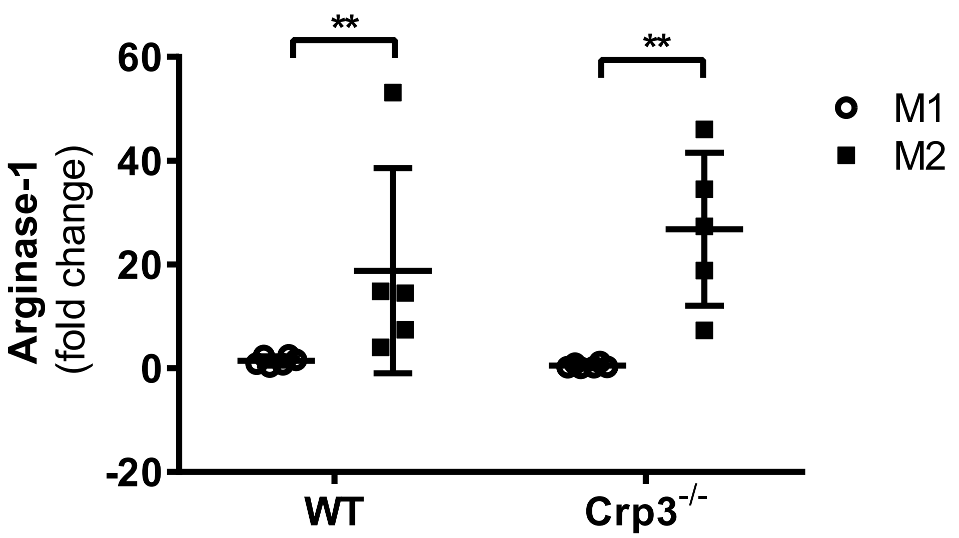

C

Gene	Forward oligonucleotide	Reverse oligonucleotide
Il1b	CCTGTGTGATGAAAGACGGC	TATGTCCCGACCATTGCTGT
Arg1	TCCAAGCCAAAGCCCATAGA	AGCTTTCCTTAATGCTGCGG

Supplementary Figure 3. Gene expression analysis for the validation of the M1 (**A**, IL-1β) and M2 (**B**, Arginase-1) macrophage phenotypes, with no difference in WT vs Crp3-/- macrophages. **(C)** Primer sequences used in the real time RT-PCR
